# Diagnostic dilemma in maxillofacial pathologies: a case series

**DOI:** 10.1186/s13256-024-04408-3

**Published:** 2024-03-21

**Authors:** Pavithra Sarda, Prashanthi Gurram, Karthik Ramakrishnan, Vivek Narayanan, Saravanan Chandran, Divya Vinayachandran

**Affiliations:** Department of Oral and Maxillofacial Surgery, SRM Kattankulathur Dental College and Hospital, SRMIST, Kattankulathur, Chengalpattu, Tamil Nadu 600203 India

**Keywords:** IGG4 disease, Secondary hperthyroidism, Kimura’s disease, Non-Hodgkin’s lymphoma

## Abstract

**Background:**

Head and neck are a site of numerous pathologies with different aetiologies and presentations. Rare pathologies, although infrequent still do exist. Diagnostic dilemma in maxillofacial pathologies can be the most challenging situation. Dealing with uncertainty, although difficult, is a reality in surgical practice. Being thorough, attentive to details and clues, and maintaining an open mind are critical strategies in the approach to such a patient.

**Case presentation:**

In our paper we are reporting a spectrum of 4 unusual variants of head and neck pathologies, whose age and sex were 52 years/ M, 37 years/F, 41 years/ F, 30 years/F respectively. All the patients were of Indian origin. The diagnosis ranged from autoimmune diseases to lymphatic cancer which posed a unique challenge both in the terms of diagnosis and management.

**Conclusion:**

A thorough systematic evaluation along with a multidisciplinary approach is mandatory in the diagnosis of unusual head and neck pathologies.

## Introduction

Head and neck region is a very complex area in the human body with various important structures. Head and neck are also a site of numerous pathologies of various aetiologies and presentations [1]. These range from infectious / inflammatory conditions to benign / malignant masses. The usual pathological enlargements in this region include orofacial infections, lymph node swellings, cystic lesions, salivary gland pathologies, thyroid masses, tumors etc.

Rare pathologies are sporadic in incidence and hence not encountered in routine practice. Though most of the surgeons are aware of these pathologies, we tend to look for the usual conditions in the first instance. When the disease does not fit the common diagnosis, it poses a challenging situation along with a diagnostic dilemma. This could be due to a number of factors: an unusual condition, an unusual presentation of a common condition, incomplete history, surgeon’s failure to recognise the disease etc.

Dealing with uncertainty, although difficult, is a reality in surgical practice. A systematic multidisciplinary approach along with a thorough clinical, radiological, histopathological examination is mandatory in the diagnosis of these swellings. Further, immunohistochemical analysis plays a vital role in differentiating rare pathologies from the common ones [2, 3, 11]. In our paper, we are reporting a spectrum of 4 unusual variants of head and neck pathologies which posed a unique challenge both in the terms of diagnosis and management.

## Case report

### Case scenario 1

**Fig. 1:**
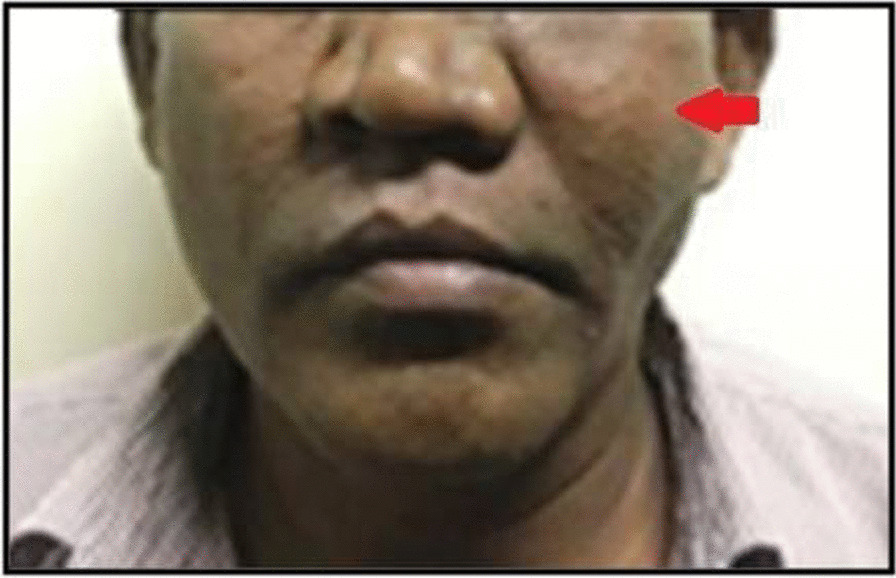
Extraoral swelling over the left premaxillary region (The arrow represents the site of swelling)

A 52-year-old male patient of Southasian (Indian) origin reported to the Department of Oral and Maxillofacial Surgery, SRM Kattankulathur Dental College and Hospital with a 3-month history of a gradually increasing swelling on the left side of the face. On local examination, a diffuse swelling was observed over the left cheek region measuring about 4 cm × 5 cm. The swelling extended superiorly from the left side infraorbital region to the commissure of the lip inferiorly, medially obliterating ala of the nose and laterally 1 cm in front of external acoustic meatus. The skin over the swelling appeared normal with no signs of infection or inflammation. The swelling was firm in consistency and non-tender (Fig. 1). On intraoral examination, a single well-defined swelling was present in the upper vestibule extending from distal surface of 23 to 27 and measuring about 4 cm × 3 cm. All the haematological investigations revealed parameters to be within the normal range. Based on the clinical findings, the provisional diagnosis made was malignancy of left maxilla.

**Fig. 2:**
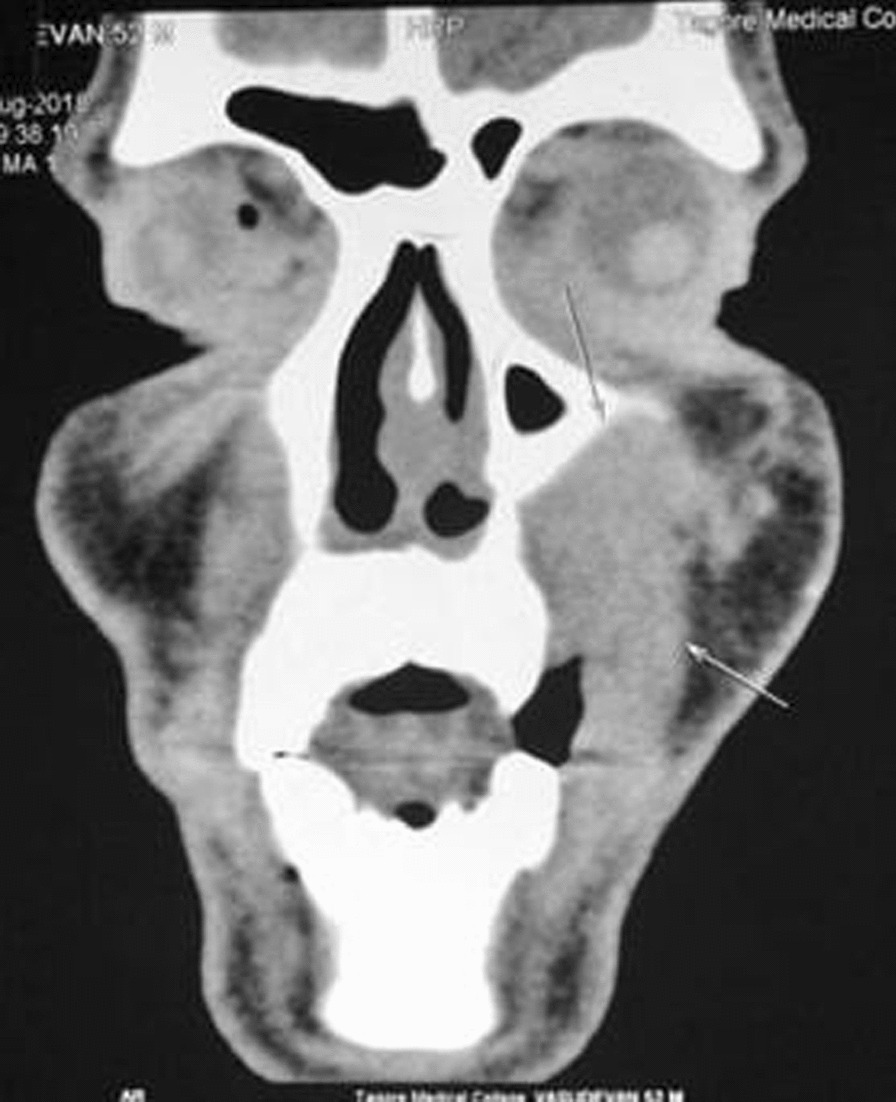
Computer tomography contrast images showing lesion over the left premaxillary region

CECT scan of head and neck showed a mild homogeneously enhancing soft tissue density in the left premaxillary region with extension and adjacent bony destruction in maxillary alveolus (Fig. 2). The differential diagnosis included benign tumor, sarcoma, lymphoma and melanoma. The incisional biopsy suggested myositis with lymphoid hyperplasia.

After complete workup, the case was taken for surgery under general anaesthesia. Tumor was excised in-toto and defect was reconstructed with a titanium mesh plate in relation to the anterior wall of the maxillary sinus and mucosal layers were closed with absorbable sutures. Soft tissue margins were sent for the frozen section intraoperatively which showed chronic inflammation with reactive fibroblastic proliferation, suggestive of inflammatory pseudotumor.

**Fig. 3:**
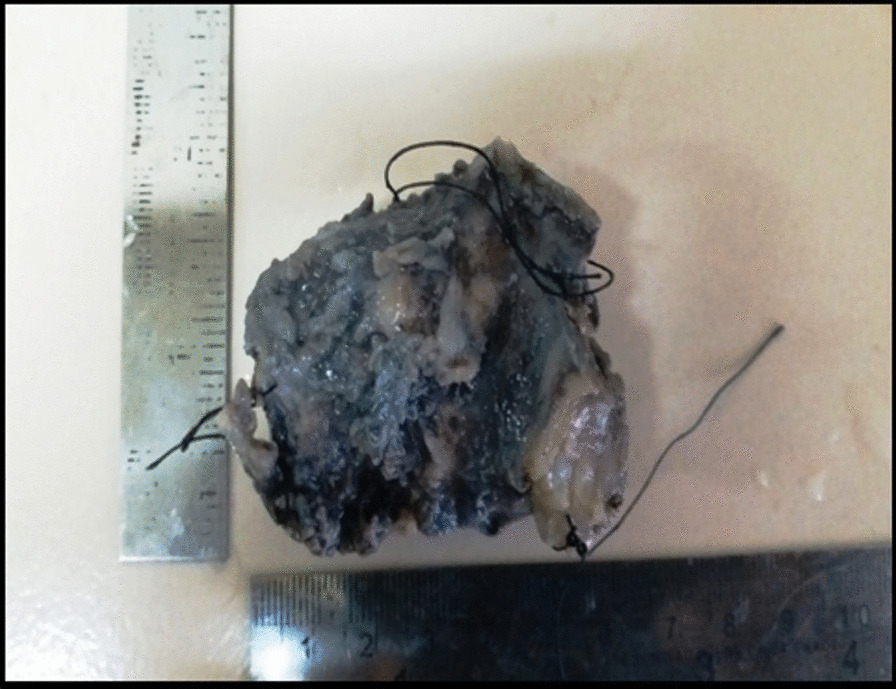
An unencapsulated gross specimen about 5cm x 5cm in size

**Fig. 4:**
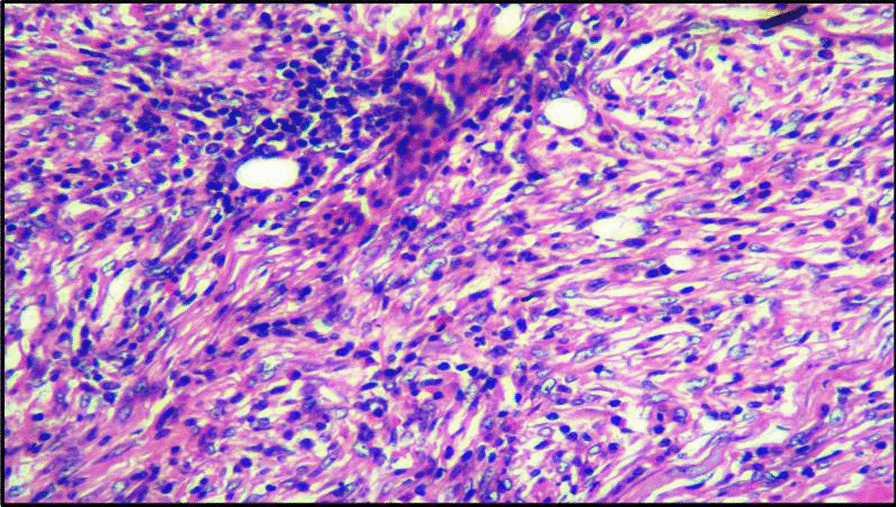
Histopathology image showing lymphoplasmocytic infiltrate with focal areas of lymphoid follicle, increased vascularity, adipocytes, and focal areas of minor salivary glands acini

On gross examination, the excised specimen showed a unencapsulated firm mass of about 5 cm × 5 cm in size (Fig. 3). The final histopathology report showed lymphoplasmocytic infiltrate with focal areas of lymphoid follicle, increased vascularity, adipocytes, and focal areas of minor salivary glands acini and suggested the diagnosis as IGG4-related disease (Fig. 4). After the uncommon final histopathology results, serum IgG4 was evaluated and found to be 0.459 g/ L (0.03–2 g /L), ESR was 44 mm (5–15 mm), and C.R.P was 9.5 mg /l (< 5 mg/ l). Hence the diagnosis was confirmed as IGG4 related disease.

Immunoglobulin G4-related disease (IgG4-RD) represents an immune-mediated fibroinflammatory condition with a characteristic histopathological appearance that can affect various organs. Because of the decreased incidence of the disease, it was challenging for a surgeon to diagnose the condition. Uchida *et al*. estimated the annual incidence of IgG4-RD at 0.28–1.08% in the 1,00,000 population [6].

### Case scenario 2

A 37-year-old female of Southasian (Indian) origin reported to our OPD with a complaint of swelling in relation to the left side of face for 1 year. Swelling was gradually progressive in size and occasionally associated with pain and history of displacement of teeth, difficulty in phonation and nasal obstruction. Patient had a history of systemic hypertension for 10 years and was under regular medication, stage V chronic kidney disease for 7 years and was under dialysis twice a week.

**Fig. 5:**
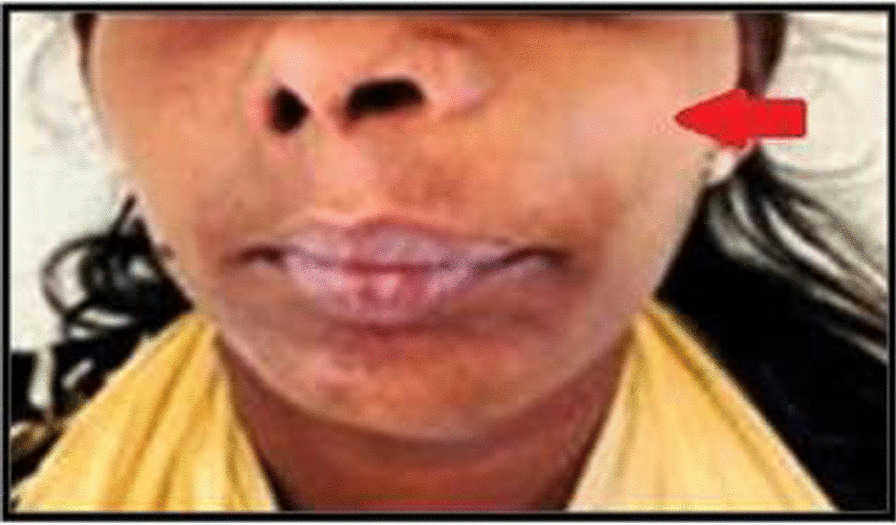
Swelling over the left side of the face (The arrow represents the site of swelling)

On local examination, a single swelling was observed measuring about 6 cm × 5 cm on the left side infraorbital region extending upto left commissure of lip, obliterating the ala of the nose medially. The skin over the swelling appeared normal, firm and non-tender (Fig. 5) Intraoral examination revealed swelling in the upper labial mucosa obliterating the vestibule extending palatally involving the complete hard palate. Mobility and spacing between the teeth was evident. Based on the medical history and clinical findings, the provisional diagnosis considered was Brown Tumor of maxilla.

**Fig. 6:**
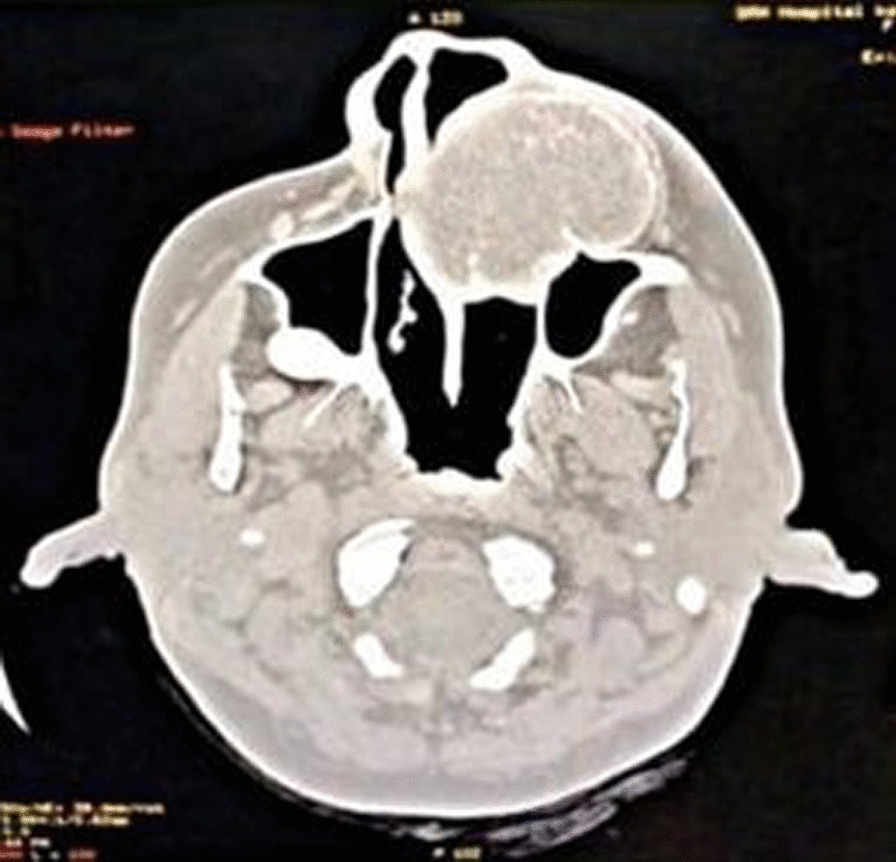
Computer tomography scan showing the lesion

**Fig. 7:**
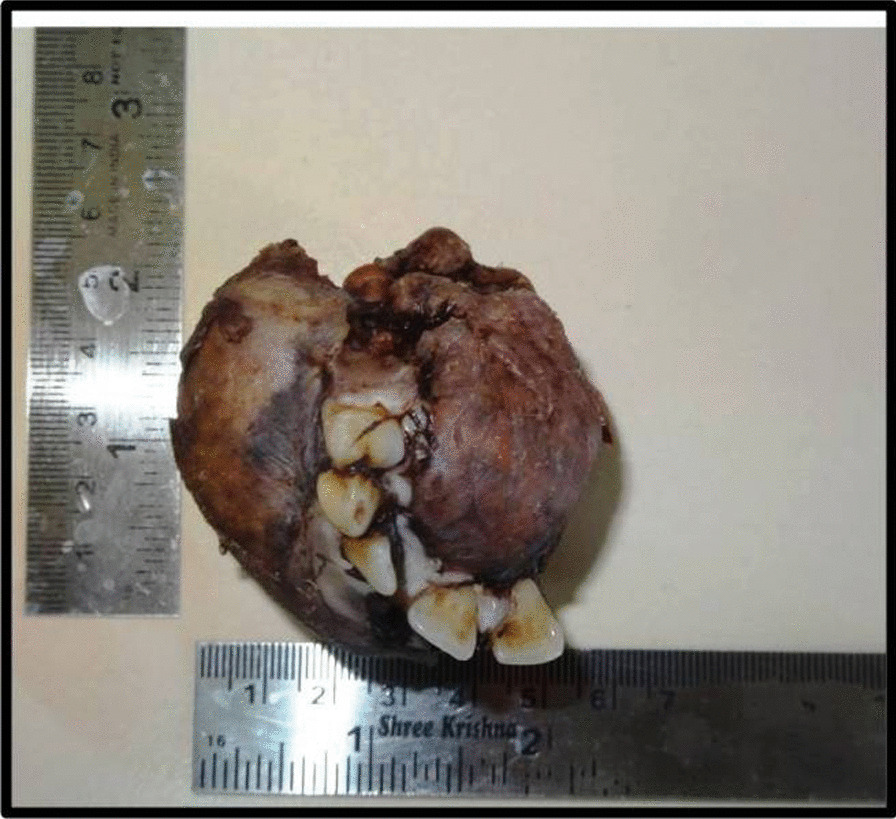
Gross examination shows a well encapsulated firm mass of 4.5 cm x 6 cm size

**Fig. 8:**
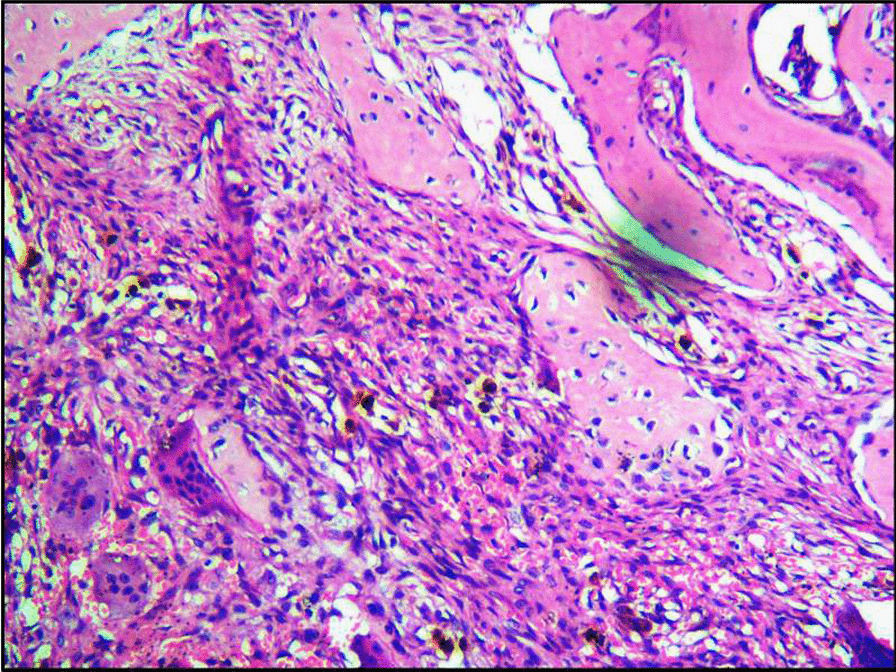
Histopathology image showing spindle cells with increased vascularity , interstitial hemorrhage and foci of hemosiderin suggestive of Brown Tumor

CT facial bones showed expansile osteolytic lesion of the left maxilla completely involving the maxillary sinus (Fig. 6). The differential diagnosis included Giant cell tumour, Ossifying fibroma, and Sarcoma. Serum chemistry revealed an elevated parathyroid hormone (PTH) level of 1423 (normal range, 15–65 pg/dl), Urea was 159 mg/dL, and Creatinine was 8.7 mg/dL. After complete workup, the patient was operated under General Anesthesia. Excision of the tumor in the anterior maxilla was done in-toto along with extraction of teeth from 21 to 25. Gross examination showed a well encapsulated firm mass of 4.5 cm × 6 cm (Fig. 7). The final histopathological report spindle cells with increased vascularity, interstitial hemorrhage and foci of hemosiderin suggestive of Brown Tumor (Fig. 8).

The final diagnosis after all the investigations was confirmed as Brown tumor of maxilla due to secondary hyperparathyroidism. Brown tumours do not represent neoplastic processes, but they are focal bony lesions due to bone remodelling from either primary or secondary hyperparathyroidism. The incidence of Brown tumors due to secondary hyperparathyroidism reported in literature is 1.5 to 1.75% [9].

### Case scenario 3

**Fig. 9:**
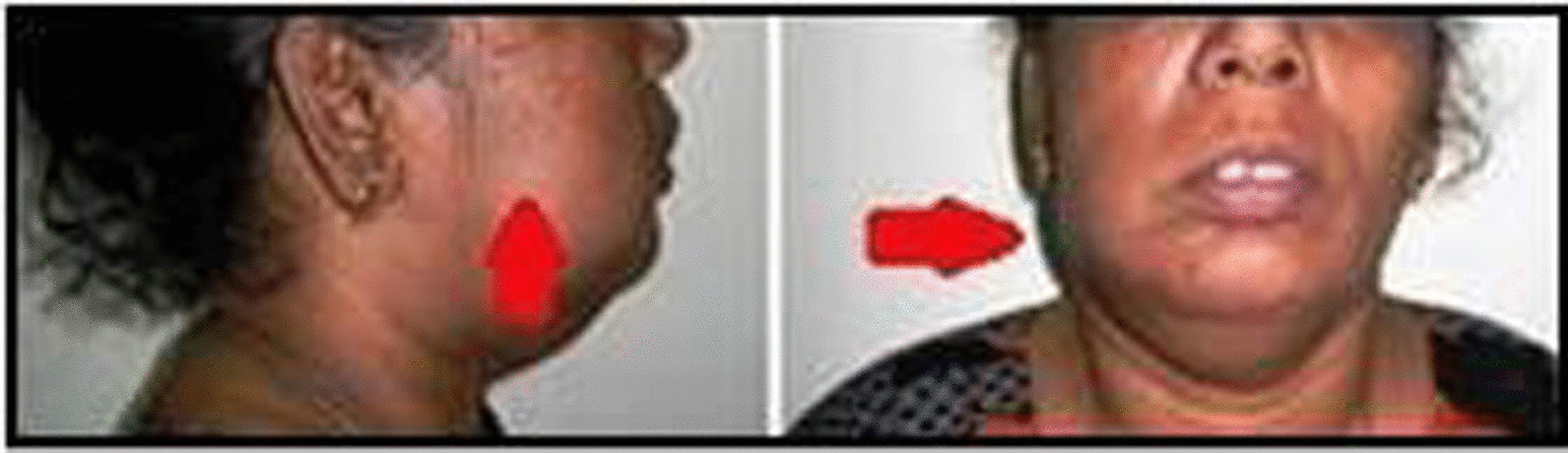
Clinical picture showing diffuse swelling over the right submandibular region (The arrows represent the site of swelling)

A 41-year-old female patient of Southasian (Indian) origin reported to our department with a 4-month history of a gradually increasing swelling on the right side of the face. The swelling was largely painless with no other associated symptoms. On local examination, a diffuse swelling was observed measuring about 6 cm × 5 cm in the right infraorbital region with regional lymphadenitis of the submandibular region of the same side. The lump was diffuse, firm, and mildly tender. Fixation to the underlying structures was not present (Fig. 9). Intraoral findings were unremarkable with no foci of infection. The haematological investigations revealed parameters to be within normal range. Based on the clinical findings, the provisional diagnosis made was Tuberculosis Lymphadenitis.

**Fig. 10:**
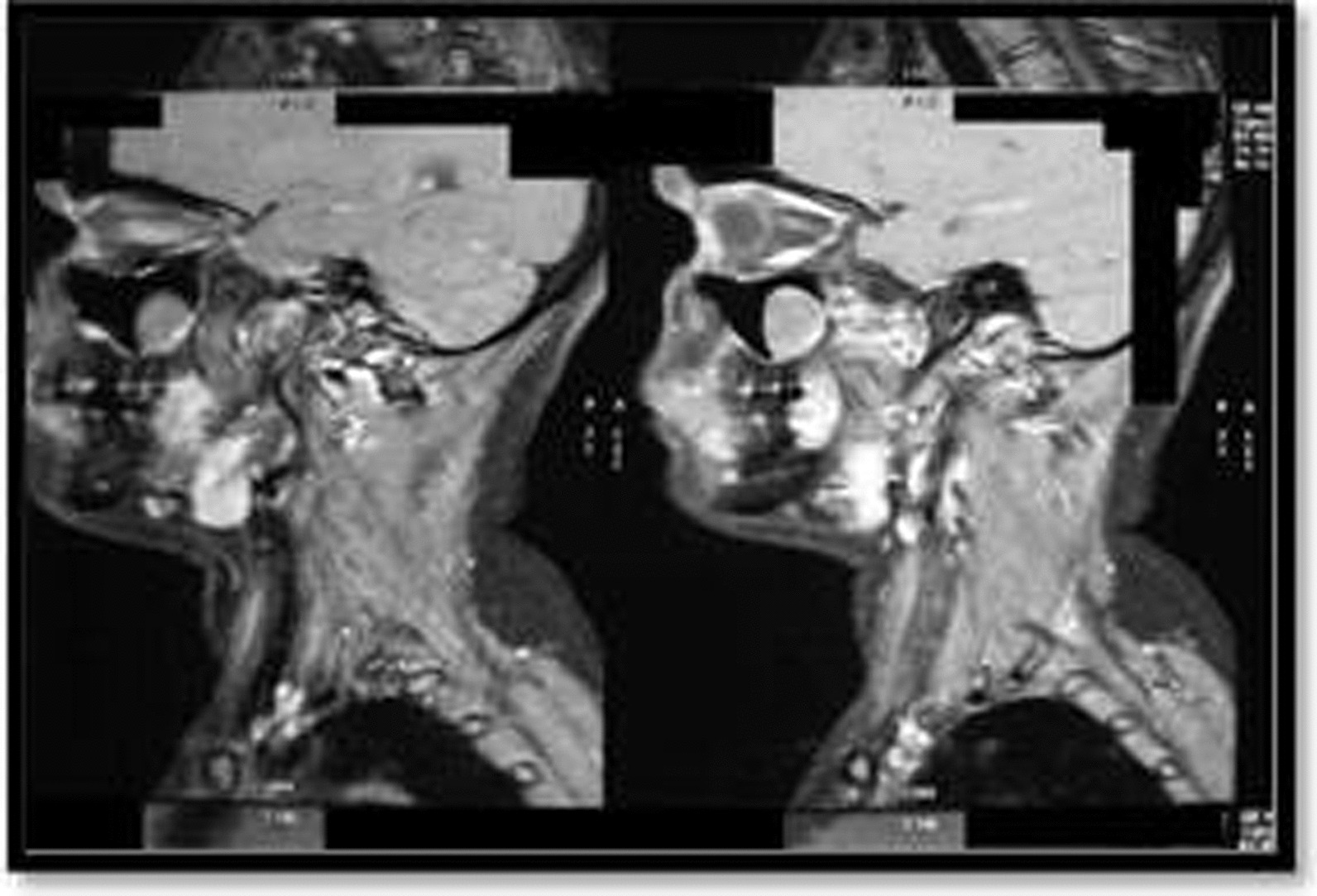
Magnetic resonance imaging (MRI) scan showing lesion over the right premaxillary region

MRI scan of the head and neck showed a homogeneously enhancing infiltrative lesion involving the premaxillary space, buccal space, retromolar trigone, and lateral pterygoid in the masticator space on the right side. Multiple enhancing solid lymph nodes were seen in the right submandibular, upper deep jugular group and the left submandibular group, largest measuring 18 mm × 12 mm in the right submandibular region (Fig. 10). MRI report provided a differential diagnosis of Neurofibroma. FNAC was performed initially, but the result was inconclusive. The incisional biopsy report suggested a nonspecific type of lymphoid hyperplasia. Definitive surgery was planned for complete excision of the lesion. Intraoperatively, it was observed that the mass was well defined, capsulated, and firm. The final histopathology report was suggestive of Reactive Lymphoid Hyperplasia with no evidence of malignancy.

The differential diagnosis comprised a wide spectrum of conditions such as Lymphoma, Fibrosarcoma, and Neuroma. Additional immunohistochemistry studies were performed to arrive at a more definitive diagnosis. It showed positivity for CD4, CD5, CD3, and CD20 and positivity for both kappa and lambda. This strongly suggested a pathology of inflammatory origin, ruling out malignancy. The patient was under close observation. One year later, the patient reported recurrent swelling in relation to the right submandibular region. FNAC was performed again, this time from the submandibular region suggesting Granulomatous Lymphadenitis. In view of the inconclusive results previously, we opted for a multidisciplinary approach to rule out other granulomatous diseases such as Atypical Tuberculosis and Sarcoidosis.

Fresh CT contrast studies were obtained. CT report suggested a diffusely enhancing soft tissue density lesion in the right buccal region involving upper and lower gingivobuccal space and retromandibular space. The region was closely abutting the right side of the mandible and obliterating the fat plane in the masticator space. Multiple enlarged lymph nodes were noted in the submandibular region and the upper deep cervical regions, the largest size being 2.1 cm in the submandibular region.

**Fig. 11:**
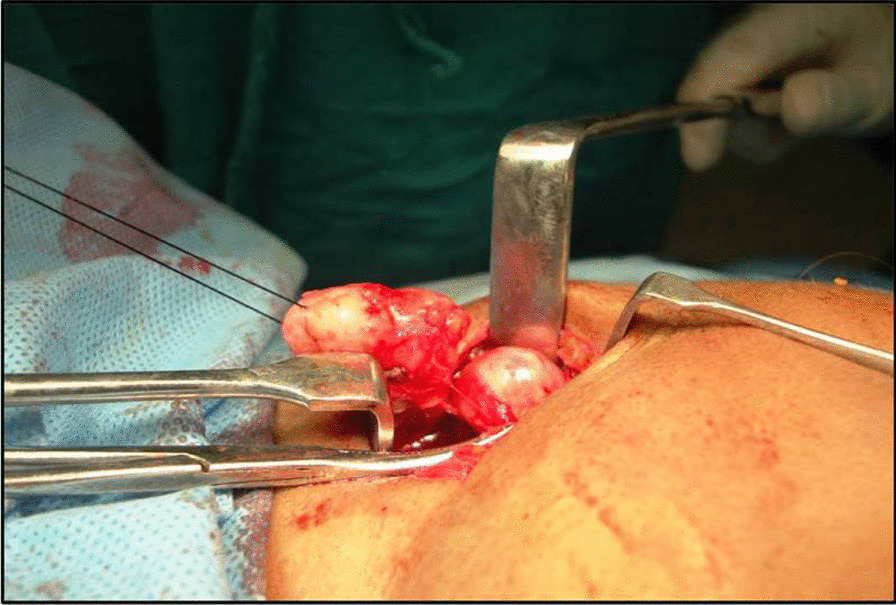
Two solid well encapsulated swellings excised intraoperatively

**Fig. 12:**
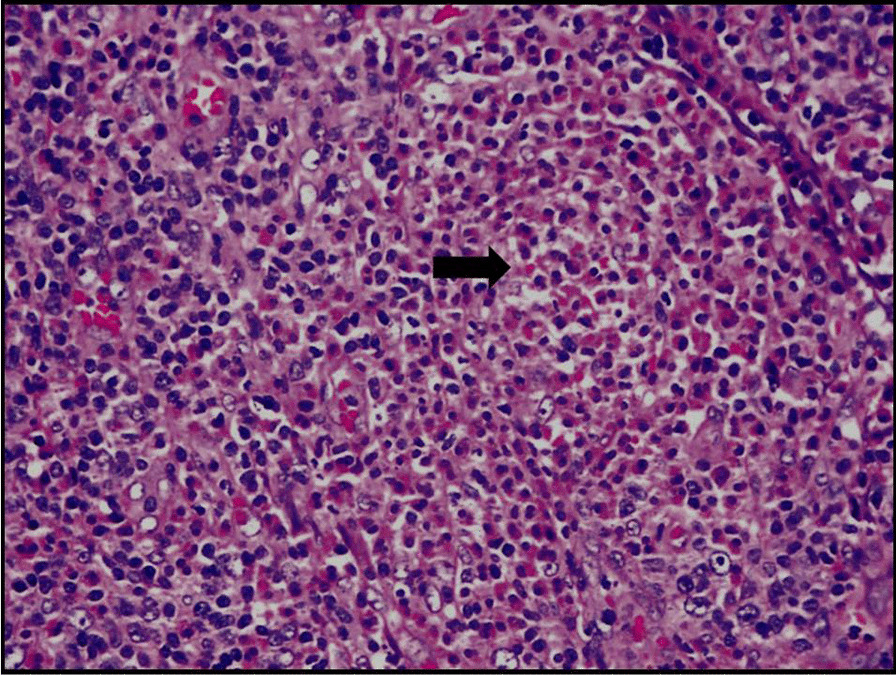
Lymph nodes with multiple predominant follicles with prominent germinal centers with extensive fibrosis and inflammatory cells including multinucleated giant cells. Arrow shows reactive follicular hyperplasia with eosinophil infiltration and penetration by venules

Lymph node biopsy was performed under general anesthesia, through a right submandibular approach. Intraoperatively, two huge solid lymph nodes, well encapsulated and firm in consistency, were removed (Fig. 11). The samples were sent for both histopathological studies and for microbiological tests. The culture test was negative for mycobacterium. The histopathology report this time suggested lymph nodes with multiple predominant follicles with prominent germinal centers with extensive fibrosis and inflammatory cells including multinucleated giant cells (Fig. 12).

Based on the histopathology report, the patient was evaluated for serum immunoglobulin E (IgE) levels, peripheral eosinophils, and absolute eosinophilic count to rule out Kimura’s disease. The IgE levels in this patient were considerably increased with 417 IU (reference value: < 180 IU), absolute eosinophil count was marginally elevated with 445/cumm (reference value: < 350/cumm), and differential count of eosinophils was 5%. The diagnosis of Kimura’s disease was confirmed thereafter based on clinical, radiological, histopathological and immunohistochemical analysis.

Kimura disease (KD) is a rare chronic inflammatory disorder with angiolymphatic proliferation, usually affecting young men of Asian race and coexisting renal disease is common. It is a rare pathology with only about 200 reported cases worldwide. It mostly seen in the second to fourth decades of life mostly in males (70–80%) [11].

### Case scenario 4

**Fig. 13:**
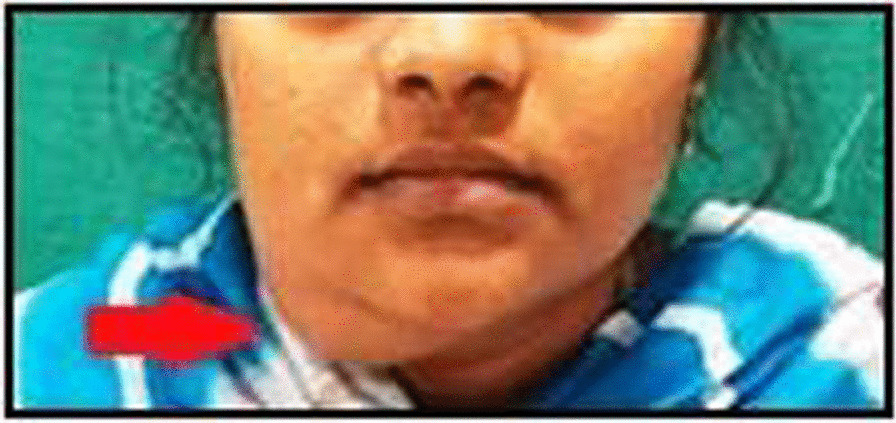
Enlargement over the right submandibular region(The arrow represents the site of swelling)

A 30-year-old female patient of Southasian (Indian) origin reported to our OPD with a 1-month history of gradually increasing swelling in the right lower region of face. On examination, a single ovoid swelling was seen along the lower border of mandible from the angle region to the midline anteriorly measuring about 3 cm × 3 cm (Fig. 13). The lump was firm and non-tender on palpation. Based on the clinical findings, the provisional diagnosis made was Submandibular Sialadenitis.

Ultrasound neck showed well defined heteroechoic lesion with peripheral increased vascularity in the right submandibular region measuring 2.4 cm × 2.0 cm × 1.9 cm suggesting lymph nodal abscess. FNAC suggested a nonspecific granuloma. MRI neck showed T2 iso to hyperintense soft tissue lesion encasing right submandibular gland likely lymph node mass—possibility of granulomatous and/ infective etiology. Patient did not report back for further treatment and opted for alternative therapy. Patient reported after 6 months with a similar chief complaint with an increase in the size of the swelling measuring about 6 cm × 4 cm and regional lymphadenitis of the submandibular region of both the sides were present. This time the skin was fixed to the underlying structures and was firm and tender on palpation.

**Fig. 14:**
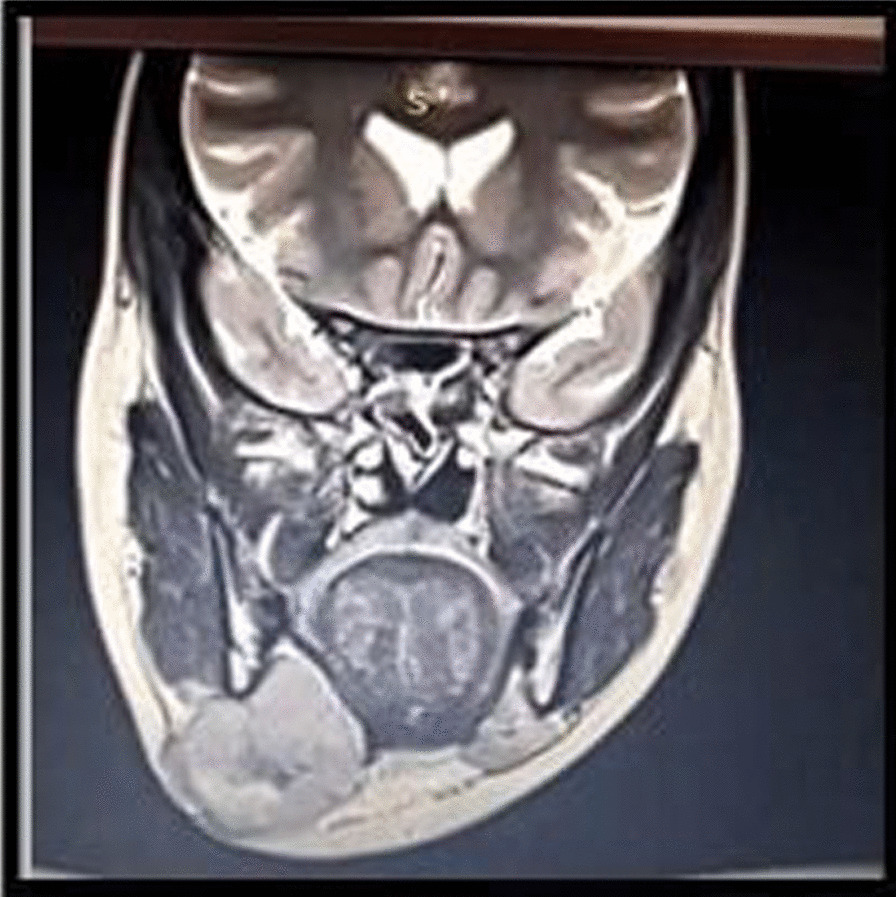
MRI scan showing well defined lobulated mass seen in the right submandibular region

Fresh MRI showed a well-defined lobulated mass is seen with its epicenter in the right submandibular region. The lesion was seen infiltrating the right submandibular gland. Perilesional inflammatory changes noted extending to the right masseter muscle, right parotid gland and the superficial subcutaneous plane. Multiple enlarged lymph nodes seen on the right in the level II/ III, and on the left side in the level IB (Fig. 14). Repeated FNAC suggested necrotizing lymphadenitis.

**Fig. 15:**
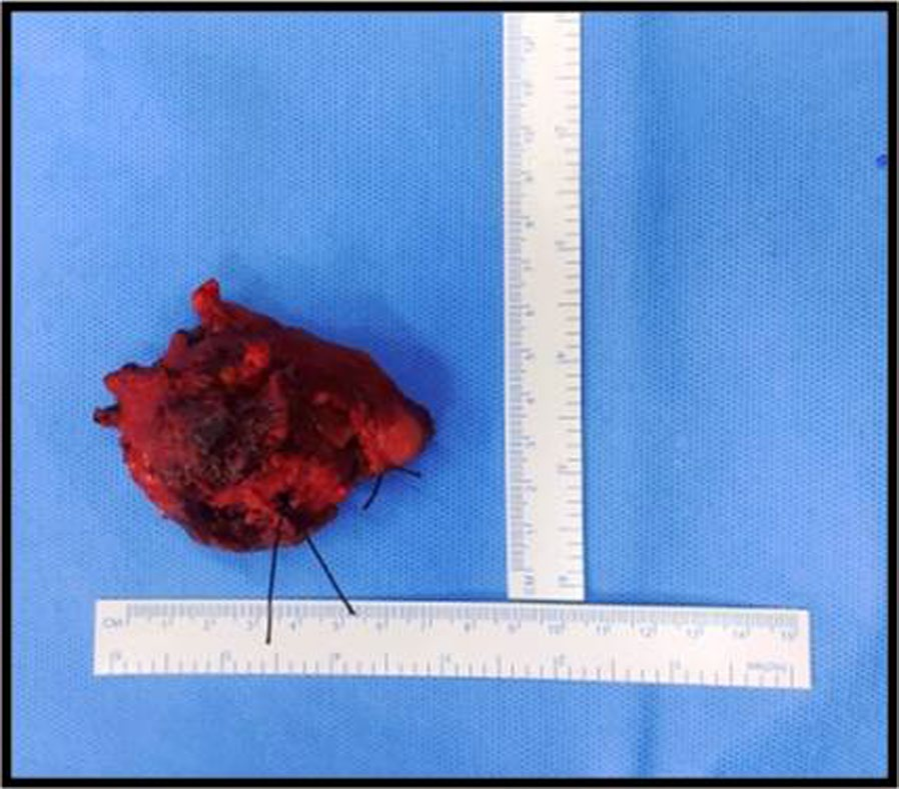
Gross specimen showing a lobulated mass measuring about 5.5 cm x 4 cm in size

The clinical differential diagnosis comprised a wide spectrum of conditions such as Plunging Ranula, TB Lymphadenopathy and Submandibular lymphadenitis. Complete work up was done and the patient was planned for wide local excision of the lesion under General anesthesia. Intraoperatively frozen section revealed no evidence of malignancy. Gross specimen showed a lobulated mass measuring about 5.5 cm × 4 cm (Fig. 15).

**Fig. 16:**
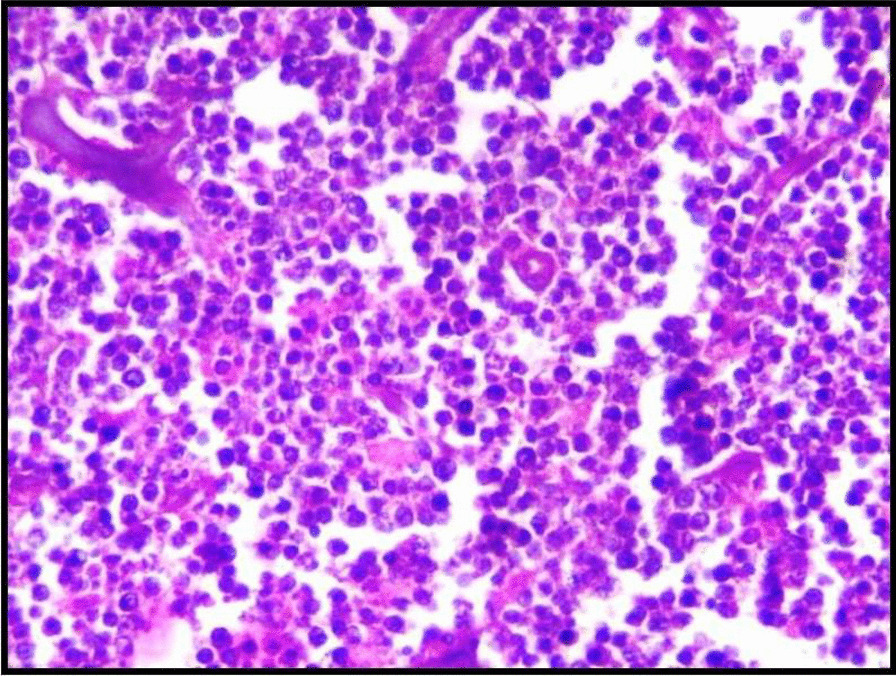
Histopathology analysis revealing atypical lymphoid cells of intermediate size

The final histopathology analysis revealed atypical lymphoid cells of intermediate size (Fig. 16). Immunohistochemistry analysis (IHC) showed atypical cells diffusely positive for CD20. Focally positive for CD45 and CD3 positive in reactive T lymphocytes. Hence this IHC analysis favoured diagnosis of Non-Hodgkin's Lymphoma-B cell type.

Lymphomas take up about 14% of all head-neck malignancies, out of which 97% are non-Hodgkin lymphomas (NHLs) with a number of extranodal onset over two times more than that of Hodgkin lymphoma. Most Non-Hodgkin lymphomas are B-cell lineage, with diffuse large B-cell lymphoma being the most commonly seen subtype, followed by small cell NHLs and Burkitt lymphoma [14].

## Discussion

Diagnostic conundrums are one of the most challenging situations for any physician or surgeon. Being thorough, attentive to clues and details, maintaining an open mind are critical strategies employed during these circumstances. The unspecific presentation of certain diseases poses a challenge for the practitioners to arrive at a specific diagnosis. In our case series, the similar problem was faced in most of the cases as there was no definite diagnosis preoperatively. Though the surgical treatment plan of the certain pathologies may be similar, if the etiology and definitive diagnosis is ambiguous, prognosis of the disease may get affected along with chances of recurrence. This is where a systematic multidisciplinary approach plays an important role in the treatment of the disease as a whole [3, 4, 11].

IgG4 is a subtype of IgG and accounts for 3–6% of the total amount of IgG. It is an increasingly recognized rare entity. It is a condition where there is inflammation and fibrosis characterized by a dense lymphoplasmacytic infiltrate rich in IgG4-positive plasma cells, storiform fibrosis and elevated serum IgG4 concentrations [5]. In our case scenario, the patient was in his fifties with no relevant medical history. The histopathology report showed lymphoplasmocytic infiltrate and changes in the level of Ig4, C-reactive protein and Erythrocyte sedimentation rate. After these specific changes, the patient was diagnosed with IgG4 related disease. The optimum treatment for this disease has not yet been established. In symptomatic patients, interventions are indicated, either through glucocorticoids and B-cell depletion to induce remission of symptoms, or through surgical interventions [6, 7].

Brown tumour are considered as unifocal or multifocal bone lesions. They appear as a serious complication of advanced hyperparathyroidism with a frequency of 1.5–1.75% in secondary hyperparathyroidism and 3–4% in primary hyperparathyroidism [9]. It usually affects young people especially females with varying degrees of aggressiveness and risks of recurrence. It is relatively rare in the maxilla with a frequency of 4.5–11.8% [9, 10]. Histopathological examination can suggest the diagnosis but it may not be sufficient to differentiate it conclusively from other lesions like giant cell lesions, ameloblastoma, aneurysmal bone cyst which can have similar microscopic and macroscopic features. A final diagnosis can be defined only by evaluating the radiological findings with histopathological, laboratory, and clinical data [10]. In our case a young female patient had a history of chronic kidney disease. After a thorough examination and various investigations, she was diagnosed with Brown tumor of maxilla due secondary hyperparathyroidism. The clinical management of a Brown tumour aims primarily to reduce the elevated parathyroid hormone levels by pharmacological management [8].

Kimura’s disease is a chronic inflammatory disorder of unknown etiology. It is postulated to be an unusual immune reaction to an unknown antigenic stimulus [12]. Our patient was a female in her forties with no relevant social history of insect bite, previous allergies, or drug-related adverse effects. There was no lymphadenopathy in any other part of the body. Her renal function was normal. Histologically, our case showed both lymph node involvement and the extranodal tissue showing marked follicular lymphoid hyperplasia with heavy eosinophilic infiltrate. Kimura’s disease can be distinguished from other diseases with tissue eosinophilia in that there is florid-reactive follicular hyperplasia, accompanied by characteristic germinal center changes [11, 13]. The diagnosis was arrived after a process of exclusion in which common causes of lymphadenitis including malignancy were ruled out. Immunohistochemical analysis finally helped confirm the disease which revealed elevated IgE levels and tissue eosinophilia. Based on the available scientific evidence, the immunologist suggested that the patient be started on systemic steroid therapy with a loading dose of 60 mg of prednisolone in divided doses for 3 months. There was a good response to steroid therapy, and the lump started to regress within 3 months which was confirmed by CT scan. Steroid dose was tapered to 2.5 mg OD over a period of 6 months. Complete remission of the lesion is observed for the past 18 months, and the patient is on a maintenance dose of 2.5 mg of oral prednisolone [11].

Non-Hodgkin’s Lymphoma (NHL) are a heterogenous group of lymphoproliferative malignancies that are much less predictable than Hodgkin’s lymphoma. In spite of all the clinical and radiological features mentioned above, none of them is specific for lymphoma, especially when the lesions are still small or solitary with a short history. Thus, patients with early NHLs are often misdiagnosed and treated with non-neoplastic diseases, such as infection and common skin diseases [14, 15]. Even in our case only after Immunohistochemistry analysis we could arrive at the confirmed diagnosis of non-Hodgkin’s lymphoma. The patient was treated with surgical excision of the lesion followed by chemotherapy.

## Conclusion

In conclusion, any persistent swelling or lump can affect both the form and function of an individual. Sometimes a multi-disciplinary means has to be chosen to aid in diagnosis and treatment of the pathology for complete cure, and to avoid further complications. A methodical approach is of prime importance not only to minimize the risk of missing the common conditions but also to consider rare possibilities.

## Data Availability

All data generated or analysed during this study are included in this published article.
